# 2D-shear wave elastography: number of acquisitions can be reduced according to clinical setting

**DOI:** 10.1186/s13244-021-01090-7

**Published:** 2021-10-21

**Authors:** Marco Dioguardi Burgio, Jules Grégory, Maxime Ronot, Riccardo Sartoris, Gilles Chatellier, Valérie Vilgrain, the group SSI-SWE, Eva Herrmann, Victor de Lédinghen, Christophe Cassinotto, Winnie C.-W. Chu, Vivian Y.-F. Leung, Giovanna Ferraioli, Carlo Filice, Laurent Castera, Jérôme Dumortier, Aymeric Guibal, Stanislas Pol, Jonel Trebicka, Christian Jansen, Christian Strassburg, Rongqin Zheng, Jian Zheng, Sven Francque, Thomas Vanwolleghem, Luisa Vonghia, Emanuel K. Manesis, Pavlos Zoumpoulis, Ioan Sporea, Maja Thiele, Aleksander Krag, Mireen Friedrich-Rust

**Affiliations:** 1 INSERM U1149 “centre de recherche sur l’inflammation”, CRI, Université de Paris, 75018 Paris, France; 2 Department of Radiology, AP-HP, Hôpital Beaujon APHP.Nord, 100 Boulevard du Général Leclerc, 92110 Clichy, France; 3 INSERM, UMR1153, Epidemiology and Biostatistics Sorbonne Paris Cité Center (CRESS), METHODS Team, Hôpital Hôtel Dieu, 75004 Paris, France; 4 Sorbonne Paris Cité, Faculté de Médecine, Université Paris-Descartes, Paris, France; 5 Assistance Publique-Hôpitaux de Paris, Hôpital Européen Georges-Pompidou, Unité de Recherche Clinique, Paris, France; 6 Centre d’Investigation Clinique 1418 (CIC1418), Paris, France

**Keywords:** Elasticity imaging techniques, Ultrasonography, Observer variation

## Abstract

**Background:**

The factors affecting intra-operator variability of two-dimensional shear wave elastography (2D-SWE) have not been clearly established. We evaluated 2D-SWE variability according to the number of measurements, clinical and laboratory features, and liver stiffness measurements (LSM).

**Methods:**

At least three LSM were performed in 452 patients who underwent LSM by 2D-SWE (supersonic shear imaging) out of an initial database of 1650 patients. The mean value of the three LSM was our best measurement method. Bland–Altman plots were used to evaluate intra-operator variability when considering only one, or the first two measurements. Variability was assessed by taking the absolute value of the difference between the first LSM and the mean of the three LSM. Logistic regression was used to assess the factors associated with the highest tertile of variability.

**Results:**

The limit of agreement was narrower with the mean of the first and second measurements than with each measurement taken separately (− 2.83 to 2.99 kPa vs. − 5.86 to 6.21 kPa and − 5.77 to 5.73 kPa for the first and second measurement, respectively). A BMI ≥ 25 kg/m^2^ and a first LSM by 2D-SWE ≥ 7.1 kPa increased the odds of higher variability by 3.4 and 3.9, respectively. Adding a second LSM didn’t change the variability in patients with BMI < 25 and a first LSM by 2D-SWE < 7.1 kPa.

**Conclusions:**

Intra-operator variability of LSM by 2D-SWE increases with both a high BMI and high LSM value. In patients with BMI < 25 kg/m^2^ and a first LSM < 7.1 kPa we recommend performing only one LSM.

**Supplementary Information:**

The online version contains supplementary material available at 10.1186/s13244-021-01090-7.

## Keypoints

- High BMI (≥ 25 kg/m^2^) and a first liver stiffness measurement by 2D-SWE ≥ 7.1 kPa were associated with higher risk of variability.
- Using the mean of two values of liver stiffness measurement decreased the variability compared to a single measurement.
- A single measurement is enough to estimate liver stiffness in non-overweight patients with a first 2D-SWE liver stiffness measurement < 7.1 kPa.

## Introduction

The staging of liver fibrosis is highly important in patients with chronic liver disease, because it influences survival and patient management. Although liver biopsy is the gold standard to assess liver fibrosis, it is associated with major limitations including rare but potentially serious complications [1] as well as a sampling variability due to the heterogeneous distribution of histological lesions [2, 3].

Liver stiffness (LS) has become a reliable noninvasive biomarker to assess liver fibrosis. The first and most widely available method is Transient Elastography (TE) [4]. TE measures the shear wave speed along one propagation line [5]. However TE has certain limitations including a lack of gray-scale image guidance, the inability to visualize and avoid large vessels and liver lesions at the measurement site, and less reliable results in obese patients and in patients with ascites [6, 7]. Unlike TE, ultrasound-based elastography can map shear wave speed or tissue stiffness in two dimensions, is guided by real time B-mode images and can be incorporated into ultrasound surveillance programs in patients with chronic liver disease. Two-dimensional (2D) shear weave elastography (SWE) based on supersonic shear imaging is an ultrasound elastography technique that has been validated in large patient populations and has been shown to have similar or better diagnostic performance than TE for the assessment of liver fibrosis [8].

Quality criteria are essential to confirm the high diagnostic value and high reproducibility of LSM. A strict acquisition protocol must be followed for all SWE techniques [7]. Moreover, the standardization of the acquisition protocol is mandatory to reduce the variability related to technical sources. In addition, the increasing of the number of measurements can also be used to reduce measure variability related to patient features. This has been defined and validated for TE and ten measurements must be obtained. However, there is no consensus on the number of measurements that should be performed using 2D-SWE. Yoon et al. proposed a minimum of six liver stiffness measurements (LSM) [9] while Choi et al. didn’t find any difference between a 5-mesurement and a 10-mesurement protocol [10] and according to other authors a 3-measurement protocol is enough [11, 12]. The recent Society of Radiologists in Ultrasound (SRU) guideline [13] recommends that at least five measures should be performed when a quality check of the measure can be assessed, while the European Federation of Societies for Ultrasound in Medicine and Biology (EFSUMB) [14] guidelines suggest a minimum of 3 measures. However the largest individual patient data-based meta-analysis evaluating LS using 2D-SWE (1650 patients), showed that the acquisition protocol was not standardized and that between one to ten measurements were performed [15].

Because ultrasound-based elastography is part of an ultrasound examination, the required number of measurements should be optimized and should take into account clinical use and diagnostic performance. Ideally, the number of acquisitions can be reduced when a technique shows high reproducibility (intra- and inter-observer agreement) and low variability [11]. The intra- and inter-observer agreement for 2D-SWE is very good [16], but can be influenced by several factors including operator experience [17] or the size of the region of interest (ROI) [18]. The influence of clinico-biological features on the variability of 2D-SWE has not been extensively studied. Preliminary results suggest that the applicability and repeatability of SWE can be affected by abdominal wall thickness, body mass index (BMI) and age [19, 20]. Nevertheless this has not been confirmed in other series [21]. Our hypothesis was that accurate identification of the clinico-biological features influencing the variability of 2D-SWE could help define and optimize the acquisition protocol according to the clinical setting.

The aims of this retrospective study were: (1) to evaluate 2D-SWE variability in a large multicenter international patient database according to the number of measurements performed, the clinico-biological features, and individual values of LSM and (2) to assess whether the number of measurements performed modified the evaluation of fibrosis stage.

## Methods

### Patient selection

The present study is an ancillary study using data extracted from the publication from Herrmann et al. [15] which complied with the “General Data Protection Regulation” requirements and was registered on clinicaltrials.gov (NCT02181452).

**Fig. 1:**
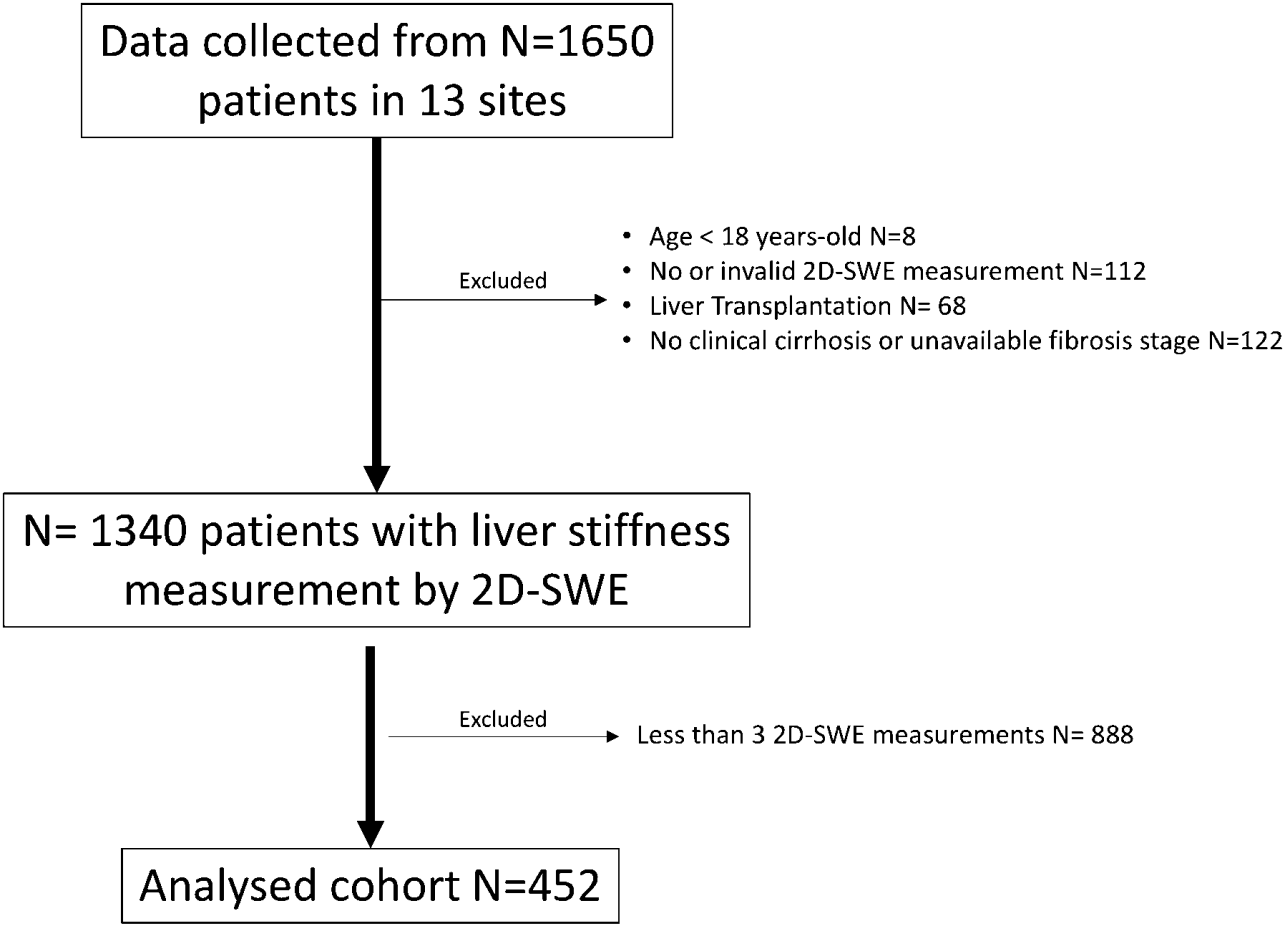
Study flowchart. 2D- SWE: two-dimensional shear wave elastography

We retrospectively analyzed a database cohort from a previous multicenter study [15] and we included all patients who met the following criteria: (1) chronic liver disease, (2) at least 3 individual LSM using 2D-SWE (this study was performed before the publication of current recommendations suggesting the minimum number of 2D-SWE acquisition to be performed [13, 14], thus large variability regarding the number of measurement (1–10) was present among the different participating centers); in case of more than three LSM, the first three were considered for analysis (3), age 18 years or older, (4) with a liver biopsy for the histological evaluation of the stage of fibrosis according to the METAVIR score (see below). We also included patients with diagnosis of cirrhosis based on a conclusive clinical examination (including a combination of ascites, jaundice, upper gastrointestinal bleeding, hepatic encephalopathy, malnutrition and cutaneous signs) and patient history. Patients who underwent liver transplantation or with less than three 2D-SWE measurements were excluded. A total of 452 patients (27.4%) from 8 sites fulfilled the inclusion criteria from an initial database of 1650 patients collected from 13 clinical centers between February 2010 and July 2014, (Fig. 1).

METAVIR stage or a comparative histological assessment was used to classify liver fibrosis as follows: none or mild fibrosis (F 0,1), significant fibrosis (F2), severe fibrosis (F3) and cirrhosis (F4) [15]. Patients with a clinical diagnosis of cirrhosis were considered to be F4. Intra-hepatic steatosis was assessed by the Brunt score [22].

### 2D-SWE technique

2D-SWE examinations were performed at all sites by senior experienced physicians blinded to the histological diagnosis. Liver biopsy was performed by experienced clinicians, and a blind histopathological analysis of biopsy samples was performed in each center.

2D-SWE was performed using the Aixplorer ultrasound system with the SC6-1 abdominal convex probe according to local practices. The technique used to perform 2D-SWE combines the generation of a remote high-amplitude shear wave using the supersonic effect applied to the acoustic radiation force, followed by an ultrafast ultrasound imaging sequence that records the propagation of the shear wave to calculate its speed in tissue [23, 24]. Tissue stiffness values (expressed in kilopascals—kPa) are displayed in real-time on a two-dimensional color-coded quantitative map, which is overlaid on the conventional grayscale B-mode image (Additional file 1: Fig. S1). 2D-SWE acquisition was performed according to the manufacturer’s instructions including: fasting (4 h), supine position, intercostal approach to the right liver lobe during a 3-s neutral breath hold. The operator adjusted the size and the location of the 2D-SWE map in the liver parenchyma depending on anatomical and clinical factors, so it was placed 1–2 cm below the liver capsule and 3–5 cm from the probe surface to homogenously cover the liver parenchyma and avoid large vessels. The liver stiffness value for each individual measurement included the mean value in the ROI.

### Statistical analysis

Our goal was to compare three methods of evaluating patients. Thus, to assess the variability, we used the mean of three consecutive LSM as our best measurement method. The alternative measurements included the first measurement alone, the second measurement alone and the mean of the first and second measurements. Pearson's correlation coefficient (*r*) and the Bland Altman plot with its 95% limits of agreement (95% LoA) were used to evaluate the agreement between these different ways of estimating the best measurement. We tested any differences between the different methods of measurement using paired t tests.

We analyzed the effect of the number of measurements on the rate of misclassifications of the staging of fibrosis (according to the METAVIR score) using one, the mean of two, or the mean of three LSM using the cut-offs proposed by Herrmann et al. [15]. Agreement was estimated using the kappa coefficient and its 95% confidence interval according to the Cohen method [25] and defined as slight (0–0.20), fair (0.21–0.40), moderate (0.41–0.60), substantial (0.61–0.80) or almost perfect (0.81–1).

Our second goal was to assess the influence of clinical characteristics on the measurement of variability. We first assessed between- and within- patient reproducibility for the measurement of fibrosis by calculating the intra-class correlation coefficients (2-way random model, R Package) and their 95% confidence intervals (CIs).

We then assessed the variability by taking the absolute value of the difference between the first LSM and the best measurement method (mean of the three LSM). We used logistic regression analysis to assess the factors that were independently associated with the highest tertile of the absolute difference between the first LSM and the average of three LSM. Variables were entered into multivariable analysis when the p value on univariate analysis was < 0.10. We have chosen to dichotomize the continuous variables to allow an easier comparison of the effect sizes of the quantitative variables with those of the qualitative variables. Due to missing values for several predictors, many observations were excluded from the multivariable analysis. BMI was the most frequently missing variable: we therefore assessed the stability of our model by performing a sensitivity analysis using a model including the same variables except the BMI.

Data are presented as mean ± SD or median (interquartile range), according to the distribution of quantitative variables and number of patients (%) for qualitative variables. The 95% confidence intervals were calculated when necessary.

A *p* value < 0.05 was considered to be significant. Statistical calculations were performed with NCSS 2020 Statistical Software (NCSS, LLC. Kaysville, Utah, USA, ncss.com/software/ncss).

## Results

### Cohort description

The final population included 452 patients, 65% men, who had three LSM with 2D-SWE (flowchart of patient selection is represented in Fig. 1). The mean age was 49.0 ± 13.5 years old. The BMI was available in 251 patients including 115 (46%) < 25, 81(32%) 25–29.9 and 55 (26%) ≥ 30 kg/m^2^.

The cause of chronic liver disease was viral hepatitis in most patients including HBV in 108/452 (24%) and HCV in 116/452 (25.5%). One hundred and fifty-one patients (33.4%) had cirrhosis. Details of the cohort are provided in Table 1. A total of 353 of these patients had a liver biopsy, while the rest had a clinical diagnosis of cirrhosis.

**Table 1:** Characteristics of the 452 patients with three 2D shear wave elastography (SWE) measurements

	*N* = 452
Age (years) ± SD (missing *N* = 29)	49.0 ± 13.5
Gender M/F (missing *N* = 18)	282(65%)/152(35%)
BMI (kg/m^2^) ± SD (missing *N* = 201)	26.3 ± 5.6
< 25 kg/m^2^	115 (46%)
25–29.9 kg/m^2^	81 (32%)
≥ 30 kg/m^2^	55 (22%)
ALD	62 (14%)
HBV	108 (24%)
HCV	116 (26%)
NAFLD	69 (15%)
Other	97 (21%)
2D-SWE registration—Biopsy delay (days) median (IQR)	0 (0; 2)
Mean LS of three 2D-SWE measures (kPa) median (IQR)	9 (6.8; 21.4)
Fibrosis stage	
F0	72 (15.9%)
F1	103 (22.8%)
F2	72 (15.9%)
F3	54 (12.0%)
F4	151 (33.4%)
Steatosis grade (missing *N* = 149)	
S0	140 (46%)
S1	82 (27%)
S2	60 (20%)
S3	21 (7%)
AST (IU/L) (missing *N* = 55) median (IQR)	48 (32; 69)
ALT (IU/L) (missing *N* = 36) median (IQR)	52 (32; 85)
Total bilirubin (µmol/L) (missing *N* = 48) median (IQR)	13 (8.6; 23.9)

*2D-SWE* two dimensional—shear wave elastography, *ALD* alcohol-related liver disease, *AST* aspartate aminotransferase, *BMI* body mass index, *CLD* chronic liver disease, *HBV* hepatitis B virus, *HCV* hepatitis C virus, *IQR* interquartile range, *LS* liver stiffness, *NAFLD* nonalcoholic fatty liver disease, *SD* standard deviation. Data are expressed as numbers (%), means (SD) or medians (IQR) as appropriate

### Bland–Altman plots

**Fig. 2:**
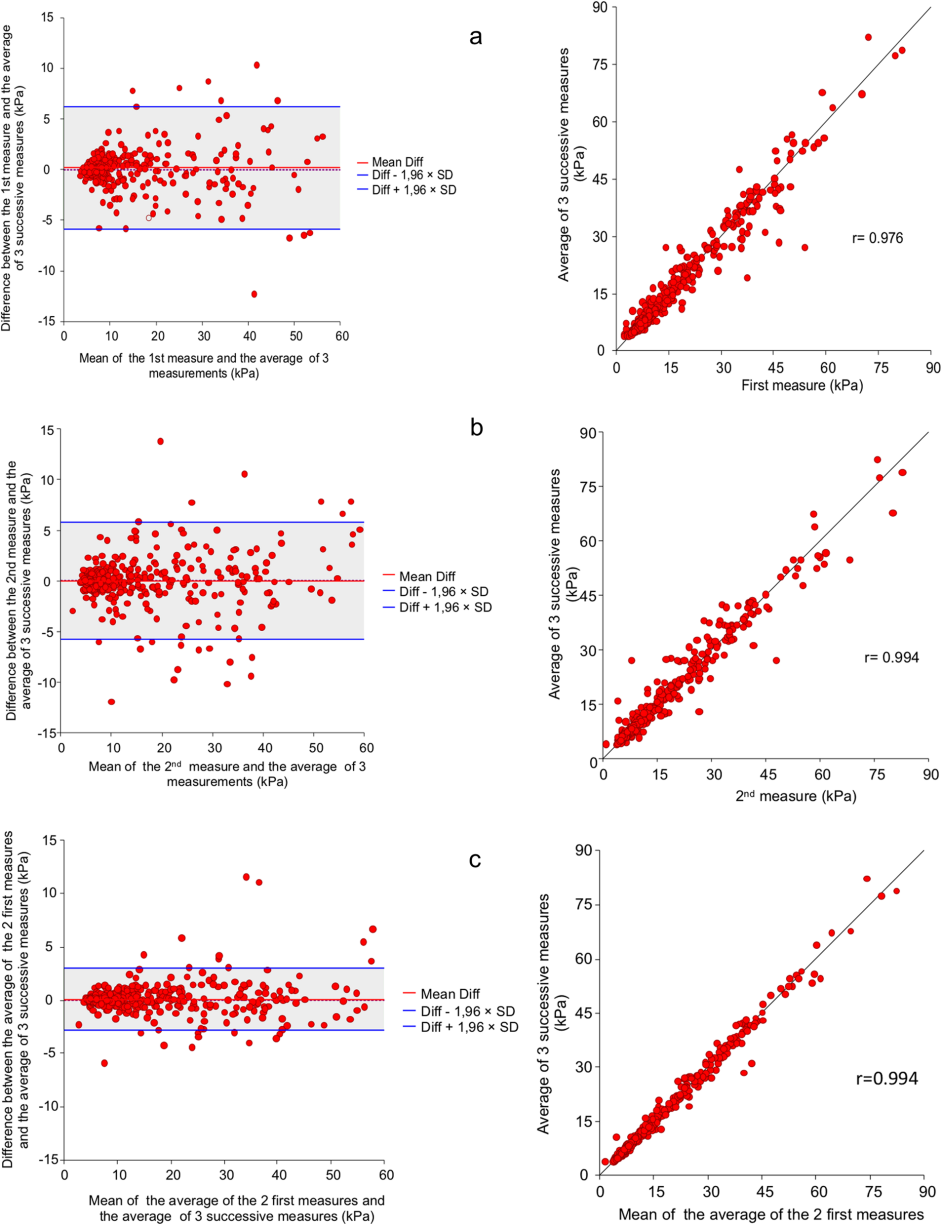
Bland–Altman and regression plots for the first liver stiffness measurement (LSM) (**a**), second LSM (**b**) and mean of first and second LSM (**c**) versus the average of the 3 consecutive LSM

When we compared the first obtained LSM to our best measurement method (mean of 3 LSM), the Bland–Altman plot showed an absence of significant measurement bias (0.17 ± 3.08 kPa). The limits of agreement (95% C.I. around the difference between the two methods of measurement) were between − 5.86 and 6.21 kPa (Fig. 2a).

When we compared the second LSM to our best measurement method (mean of 3 LSM) we also noted an absence of significant bias (− 0.02 ± 2.9 kPa) and the limits of agreement were between − 5.77 and 5.73 kPa (Fig. 2b).

The mean of the first and second measurements was associated with a persistent absence of bias (0.08 ± 1.48 kPa) but with a narrower limit of agreement than the two single measurements (− 2.83 to 2.99 kPa—Fig. 2c).

### Influence of the number of LSM on fibrosis classification

Kappa values showed moderate to substantial agreement between LSM and the stage of fibrosis. The kappa value was more dependent on the method of classification of the stage of fibrosis than on the number of measurements which had almost no influence (Table 2).

**Table 2:**
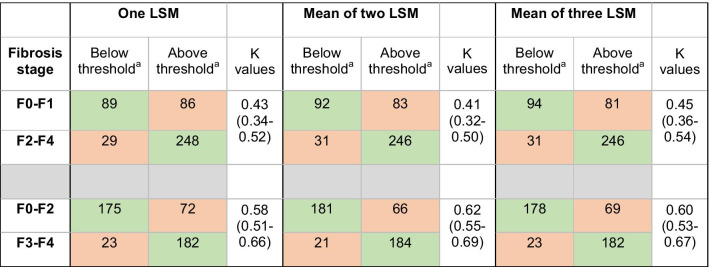
Classification of 452 patients for the stage of fibrosis grade to one, the mean of two or the mean of three liver stiffness measurement using 2D shear wave elastography

^a^Threshold for LS measure was 7.1 kPa for differentiation of F0–1 versus F2–4 and 9.2 kPa (except for HBV-related liver disease where a cutoff of 8.2 kPa was used) for differentiation of F0–2 versus F3–4, according to Herrmann et al. (15); LSM: liver stiffness measurement

The appropriate classification is represented with a light green background, while the misclassification is represented with a light red background

#### F0–F1 versus F2–F4

Using only one LSM led to a misclassification of the stage of fibrosis in 115/452 (25.4%), including 29 cases of underestimation and 86 cases of overestimation. When the mean of two or three LSM was used, the misclassification rate was similar (25.2% and 24.7% respectively—Table 2).

#### F0–F2 versus F3–F4

The use of only one LSM led to the misclassification of the stage of fibrosis in 95/452 (21%) patients, including 23 cases of underestimation and 72 cases of overestimation. When the mean of two or three LSM was used, the misclassification rate was similar (19.2% and 20.3% respectively—Table 2).

### Variability according to the intra-class correlation and the absolute value of the difference between the first LSM and the mean of three LSM

The ICC calculated from the three LSM in each patient was 0.93 [0.92–0.94] for the whole population. Changes in the ICC according to clinical, biological and pathological features are set out in Additional file 1: Table S1. ICC values decreased as the BMI increased.

Analysis of tertiles for the absolute value of the difference between the first LSM and the mean of three LSM are set out in Table 3. Several factors were more frequently associated with a higher variability in 2D-SWE measurements (upper tertile): age > 50, BMI > 25 kg/m^2^, serum AST ≥ 50 IU/L, serum total bilirubin ≥ 20 µmol/L, alcohol intake as the etiology of chronic liver disease, the presence of cirrhosis, and high LSM value on 2D-SWE.

**Table 3:** Characteristics of patients according to the tertiles of the absolute value of the difference between 1 (first acquisition) and the mean value of 3 liver stiffness measurements

	Low tertile	Central tertile	High tertile	*p*
Gender (*N* = 434)				
Male	81 (28.7%)	97 (34.4%)	104 (36.9%)	0.312
Female	48 (31.6%)	59 (38.8%)	45 (36.9%)	
Age (years) (*N* = 423)				
< 50	69 (35.6%)	76 (39.2%)	49 (25.2%)	< 0.01
> 50	54 (23.6%)	78 (34.0%)	97 (42.4%)	
BMI kg/m^2^ (*N* = 251)				
< 25	49 (42.6%)	44 (38.3%)	22 (21.7%)	< 0.01
25–30	10 (12%)	26 (32%)	45 (56%)	
≥ 30	10 (18%)	15 (27%)	30 (55%)	
AST (IU/L) (*N* = 397)				
< 50	89 (42.2%)	69 (32.7%)	53 (25.1%)	< 0.01
≥ 50	39 (21%)	68 (36.5%)	79 (42.5%)	
Total bilirubin (µmol/L) (*N* = 404)				
< 20	109 (38.6%)	109 (38.6%)	64 (22.8%)	< 0.01
≥ 20	24 (19.7%)	32 (26.2%)	66 (54.1%)	
Cause of CLD				
ALD	6 (9.7%)	17 (27.4%)	39 (62.9%)	
HBV	60 (56%)	28 (26%)	20 (18%)	
HCV	36 (31%)	43 (32%)	37 (32%)	< 0.01
NAFLD	17 (24.6%)	23 (33.3%)	29 (42%)	
Other	31 (32%)	41 (42%)	25 (26%)	
Fibrosis stage				
F0	37 (51%)	20 (28%)	15 (21%)	
F1	46 (44.5%)	45 (44.5%)	12 (11%)	
F2	30 (42%)	27 (37%)	15 (21%)	< 0.01
F3	18 (33%)	20 (37%)	16 (30%)	
F4	16 (11%)	44 (29%)	91 (60%)	
Steatosis (303)				
S0	41 (29.3%)	56 (40.0%)	43 (30.7%)	
S1	20 (24.4%)	36 (43.9%)	26 (31.7%)	0.707
S2	18 (30.0%)	24 (40.0%)	18 (30.0%)	
S3	9 (42.9%)	5 (23.8%)	7 (33.3%)	
First LSM				
< 7.1 kPa	77 (62.6%)	38 (30.9%)	8 (6.5%)	< 0.01
≥ 7.1 kPa	70 (21.3%)	118 (35.9%)	141 (42.9%)	
First LSM				
< 9.2 kPa	114 (54.2%)	73 (34.8%)	23 (11.0%)	< 0.01
≥ 9.2 kPa	33 (13.6%)	83 (34.3%)	126 (52.1%)	
First LSM				
< 13 kPa	128 (46.7%)	106 (38.7%)	40 (14.6%)	< 0.01
≥ 13 kPa	19 (10.7%)	50 (28.1%)	109 (61.2%)	

*ALD* alcohol-related liver disease, *AST* aspartate aminotransferase, *BMI* body mass index, *CLD* chronic liver disease, *HBV* hepatitis B virus, *HCV* hepatitis C virus, *LSM* liver stiffness measurement, *NAFLD* nonalcoholic fatty liver disease

Factors independently associated with a higher tertile of variability are shown on multivariable logistic regression analysis (Table 4). A BMI ≥ 25 kg/m^2^ and the first LSM ≥ 7.1 kPa increased the odds of a higher variability by 3.4 and 3.9, respectively. Both viral and other non-alcoholic causes of chronic liver disease were associated with a lower variability than alcohol-related chronic liver disease.

**Table 4:** Influence of various patient characteristics on the variability of measurements estimated as the absolute difference between the first liver stiffness measurement and the mean of 3 liver stiffness measurements. Multivariable logistic regression analysis predicting the upper tertile of variability

Variable	Odds ratio	Lower 95% CI	Upper 95% CI	*Z* value	*P* value
Age ≥ 50 years	0.966	0.486	1.919	− 0.099	0.9212
BMI ≥ 25 kg/m^2^	3.405	1.788	6.483	3.729	0.0002
Etiology					
Viral versus alcohol	0.371	0.149	0.921	− 2.137	0.0326
Other versus alcohol	0.651	0.235	1.808	− 0.823	0.4103
AST ≥ 50 (IU/L)	0.910	0.466	1.774	− 0.278	0.7812
Bilirubin ≥ 20 (µmol/L)	2.069	1.005	4.257	1.974	0.0484
LSM ≥ 7.1 kPa	3.928	1.583	9.744	2.951	0.0032

*AST* aspartate aminotransferase, *BMI* body mass index, *LSM* liver stiffness measurement

When BMI (which represented most of the missing values) was not included in the model no differences in either variable selection or effect sizes were observed (Additional file 1: Table S2).

**Fig. 3:**
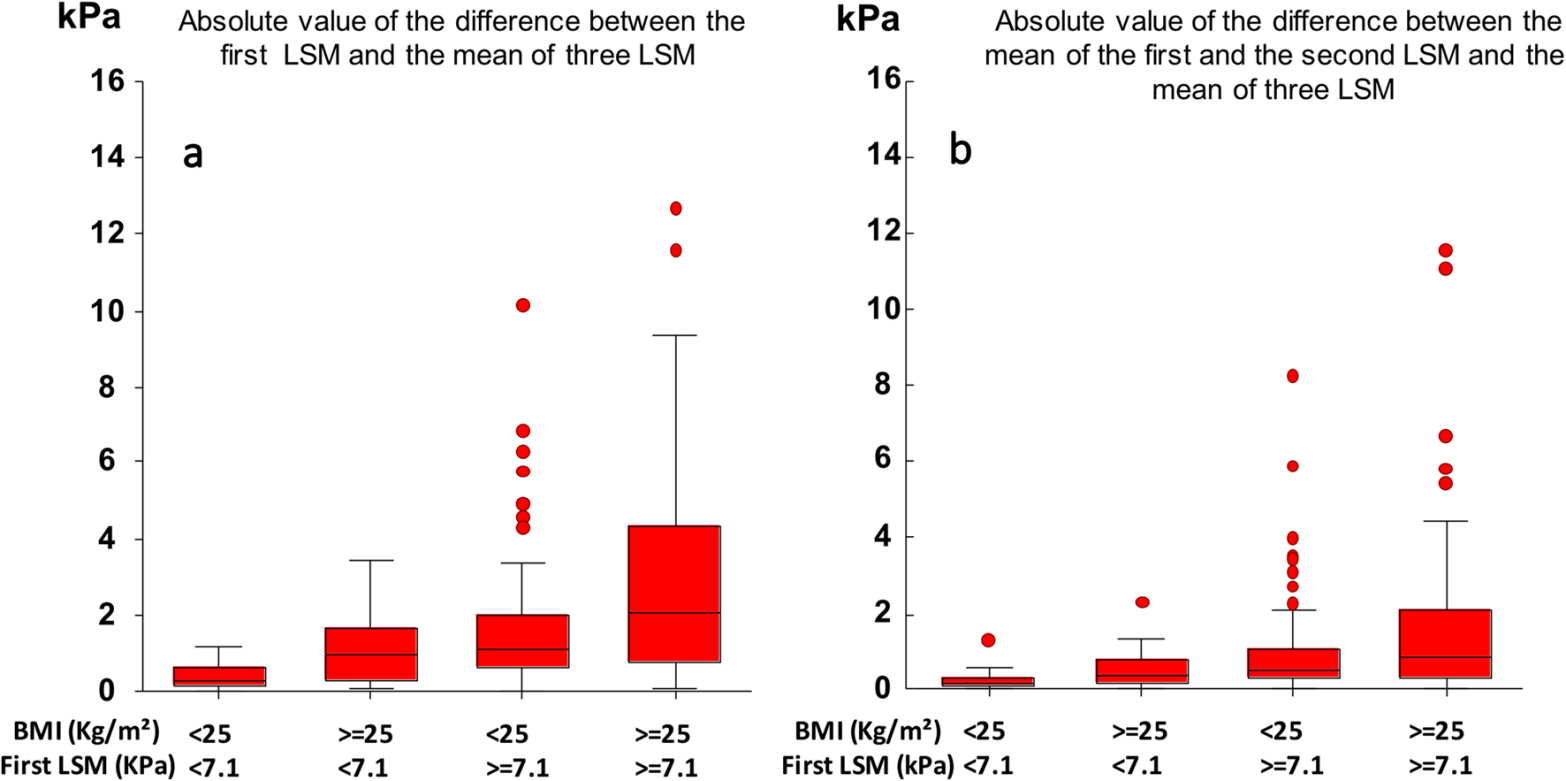
Variability of the first (**a**) liver stiffness measurement (LSM) and of the mean of the first and second (**b**) LSM according to body mass index (BMI) and the value of liver stiffness obtained by the first measurement using two-dimensional shear wave elastography

A low variability in 2D-SWE (absolute value of the difference between the first LSM and the mean of three LSM) was found in patients with a BMI < 25 kg/m^2^ and a first LSM < 7.1 kPa (median variability: 0.25 kPa Fig. 3a). The variability in 2D-SWE measurements remained unchanged in these patients when a second measurement was added (median variability 0.2 kPa) (Fig. 3b).

Conversely, the variability was higher in patients with a BMI ≥ 25 kg/m^2^ and the first LSM ≥ 7.1 kPa (median variability 2 kPa—Fig. 3a). In these patients, the variability decreased when a second measurement was added (median variability: 0.87 kPa) (Fig. 3b).

## Discussion

This study showed that performing two LSM with 2D-SWE reduced the variability compared to a single measurement. Variability in 2D-SWE is influenced by several patient-related factors, in particular the BMI and the value of the first LSM. Our results suggest that in non-overweight patients, when an initial LSM < 7.1 kPa is obtained, a single measurement is sufficient to estimate LS.

Unlike TE, there is no strict consensus on the number of LSM that should be performed with 2D-SWE per patient or whether this could be reduced in relation to the clinical setting. A study by Choi et al. [10] did not report any difference in LS values using a 5-measurement or a 10-measurement protocol. Interestingly, two recent meta-analyses [15, 26] confirmed the heterogeneity of the number of acquired LSM using 2D-SWE in clinical practice. Indeed, even in expert tertiary centers, the number of measurements performed per patient varied, with most of the teams using 3- or 5- measurement protocols. Some differences also exist according to consensus guidelines, the EFSUMB guidelines suggest that a minimum of three LSM measures [14] while a minimum of five LSM measurement is recommended by the SRU guidelines [13]. While a 3 to 5 measurement protocol is a good compromise to obtain reliable measurements in research, our goal was to evaluate simpler and quicker acquisitions with a “low risk of variability” that could facilitate the use and the spread of ultrasound-based LSM in daily clinical practice [11], beyond a research setting.

Overall, when the average of three LSM measurements was used as our reference value, we found that the mean value of two LSM decreased measurement variability compared to a single measurement. This was seen by a narrower limit of agreement in Bland–Altman plots, suggesting that at least two LSM should be performed with 2D-SWE to reduce variability without taking into consideration the clinical factors. We also looked at the number of LSM and the misclassification rate of fibrosis stage. Interestingly, in our cohort, the rate of misclassification of the stage of fibrosis was similar with two or three LSM and a single measurement, confirming the overall good intra-operator reproducibility of LSM with 2D-SWE.

Considering that the use of a strict standardized acquisition protocol for each LSM allowed us to minimize the effect of variability related to technical sources, to identify the patient-related factors associated with a greater variability in 2D-SWE, we performed multivariable analysis and showed that high LSM values increase the variability of 2D-SWE. We evaluated a threshold of 7.1 kPa which is clinically relevant because it is the optimal cut-off to distinguish patients with significant (F2-4) fibrosis from those without [15]. For example, an initial LSM > 7.1 kPa increased the odds of greater variability by 3.9. This is supported by the results from a study by Choi et al. [10] which showed that variability in 2D-SWE increases with LS values > 10 kPa. This also confirms our observation that the 2D-SWE color map tends to be more heterogeneous in daily clinical practice, with high LS values.

The second patient related factor in our cohort that increased 2D-SWE variability was the BMI. On multivariable analysis a BMI > 25 kg/m^2^ increased the odds of high 2D-SWE variability by 3.4 times. The BMI is known to reduce reproducibility of TE [27] and to increase inter-modality disagreement for the assessment of fibrosis in patients with chronic HCV [28, 29].

Discordant results have been reported in the literature for 2D-SWE. Hudson et al. [30] showed that a BMI of more or less than 25 kg/m^2^ did not influence the reproducibility of LSM. Nevertheless, this study was performed in healthy volunteers. Mancini et al. [21] reported that BMI didn’t influence inter-observer agreement of SWE, but the cohort was small (29 patients) and a different US platform was used in this study. Similar results were reported by Mulabecirovic et al. [31]. In contrast, in our cohort a BMI ≥ 25 kg/m^2^ was found to be strongly associated with greater variability. The increased variability of LSM in overweight and obese patients is thought to be related to the increased thickness of the abdominal wall [19]. In the study by Yoon et al. [32] waist circumference had a high diagnostic value (AUC 0.82) for the prediction of inter-observer disagreement in fibrosis stage on 2D-SWE. Moreover, there was also a risk of inter-observer disagreement in patients with a BMI > 23.8 kg/m^2^ and a waist circumference > 84 cm. The meta-analysis by Kim et al. [26] also showed that a high BMI and obesity were factors affecting variability, and that they are the most frequent parameters influencing technical failures or unreliable LSMs with 2D-SWE.

Interestingly, in our study, 2D-SWE variability was very low in patients with a BMI < 25 kg/m^2^ and a first LSM < 7.1 kPa. Moreover, adding a second LSM didn’t modify 2D-SWE variability in these patients. Thus, our results suggest that if a first LSM < 7.1 kPa is obtained in non-overweight patients, a single measurement is sufficient to correctly estimate LS with a very low risk of variability and misclassification.

Our study has several limitations. First, the retrospective design from a multicenter database may result in a selection bias. However, all acquisitions were performed by skilled operators following a standard protocol [15]. Although certain data were missing because of the retrospective design such as BMI, results remained consistent when BMI was excluded from the multivariable analysis. We used a three LSM protocol as a best measurement method for the evaluation of variability, which is below the five LSM protocol suggested by the SRU guidelines.

No manufacture-driven quality criteria (i.e. stability index) nor interquartile range values were available at the time the acquisition was performed. Therefore, some unreliable LSM values could have been included in our population. Nevertheless, this is probably compensated by the large sample of population and by the multicenter setting including only tertiary centers. The effect of LSM variability according to the recruiting centers was not explored, which could be another source of bias. Yet, we did not test such possible variability as the distribution of patient population (liver diseases and degree of liver fibrosis) and the distribution of missing data were also variable according to centers. Finally, whether our result could be generalized to other US platforms should be explored in dedicated studies, nevertheless intersystem agreement is demonstrated to be good to excellent in both phantom and in-vivo acquisitions between different 2D-SWE systems [33].

In conclusion, intra-observer variability of LSM with 2D-SWE based on supersonic shear imaging can be influenced by several patient-related factors, in particular high BMI and LS values. Our study shows that performing two LSM instead of one decreased the variability of LS values with only a slight impact on the misclassification of the stage of fibrosis. Because of the low variability of LSM in patients who are not overweight and have an initial LSM < 7.1 kPa, a single measurement should be sufficient to estimate LS in these patients. We think that a shorter 2D-SWE acquisition protocol could be used in selected patients to extend ultrasound-based elastography from research to daily clinical practice.

## Supplementary Information

**Additional file 1.** Supplemental tables and supplemental figure.

## Data Availability

The datasets used and/or analyzed during the current study are available from the corresponding author on reasonable request.

## Abbreviations

BMI: body mass index
LS: liver stiffness
LSM: liver stiffness measurement
ROI: region of interest
SWE: shear wave elastography
TE: transient elastography
