# Supplementary material for: 2D-shear wave elastography: number of acquisitions can be reduced according to clinical setting
Source: Insights Imaging. 2021 Oct 21;12:145. doi: 10.1186/s13244-021-01090-7 (PMC8531167; doi:10.1186/s13244-021-01090-7)

**Additional file 1**

**Supplemental Table 1**

Influence of various patients’ characteristics on the variability of measurement estimated by the intra-class correlation coefficient (ICC)

| Variable | ICC value (95% CI) |
| --- | --- |
| Age (years)  <50  ≥50 | 0.91 (0.90; 0.93)  0.93 (0.91; 0.94) |
| Gender  Male  Female | 0.95 (0.94; 0.96)  0.89 (0.87; 0.91) |
| BMI (Kg/m^2^) <25 25-30 ≥30 | 0.97 (0.96; 0.98)  0.89 (0.85; 0.92)  0.86 (0.81; 0.91) |
| Cause of CLD  HBV HCV NAFLD ALD Other | 0.95 (0.94; 0.97)  0.91 (0.89; 0.93)  0.86 (0.82; 0.90)  0.92 (0.89; 0.95)  0.92 (0.90; 0.94) |
| METAVIR Fibrosis stage  F0-F1 F2–F4 | 0.87 [0.84; 0.90]  0.92 [0.91; 0.94] |
| Brunt Steatosis Score  S0 S1 S2 S3 | 0.94 [0.93; 0.96]  0.92 [0.89; 0.94]  0.86 [0.81; 0.91]  0.91 [0.85; 0.95] |
| AST (IU/L)  <50 ≥50 | 0.91 [0.90; 0.93]  0.94 [0.93; 0.95] |
| Total bilirubin (µmol/L)  <20 ≥20 | 0.89 [0.87; 0.90]  0.92 [0.90; 0.94] |

ALD: alcohol-related liver disease; AST: aspartate aminotransferase; BMI: body mass index; CLD: chronic liver disease; HBV: hepatitis B virus; HCV: hepatitis C virus: NAFLD: nonalcoholic fatty liver disease

**Supplemental table 2**

Influence of various patients’ characteristics on the variability of measurement estimated as the absolute difference between the 1^st^ liver stiffness measurement and the mean of 3 measurements. Multivariable logistic regression analysis predicting the upper tertile of variability. Patients without BMI value (missing values) were excluded.

| Variable | Odds Ratio | Lower 95% CI | Upper 95% CI | Z-Value | P Value |
| --- | --- | --- | --- | --- | --- |
| Age ≥ 50 years | 1.37879 | 0.82848 | 2.29462 | 1.236 | 0.21647 |
| Etiology: |  |  |  |  |  |
| Viral vs. alcohol | 0.42864 | 0.20798 | 0.88342 | -2.296 | 0.02168 |
| Other vs. alcohol | 0.61651 | 0.29132 | 1.30472 | -1.265 | 0.20603 |
| AST ≥ 50 (IU/L) | 1.19699 | 0.71805 | 1.99537 | 0.690 | 0.49043 |
| Bilirubin ≥ 20 (µmol/L) | 1.93390 | 1.12164 | 3.33437 | 2.373 | 0.01765 |
| LSM ≥7.1 kPa | 5.55707 | 2.50476 | 12.32891 | 4.218 | 0.00002 |

AST: aspartate aminotransferase; LSM: liver stiffness measurement

**Supplemental Figure 1.**2-dimensional shear wave elastography acquisition in a 63-year-old patient with liver cirrhosis. Tissue stiffness values (expressed in kilopascals - kPa) are displayed in real-time on a two-dimensional color-coded quantitative map overlaid on the conventional grayscale B-mode image. The circle within the colour map represents the region of interest chosen by the radiologist.


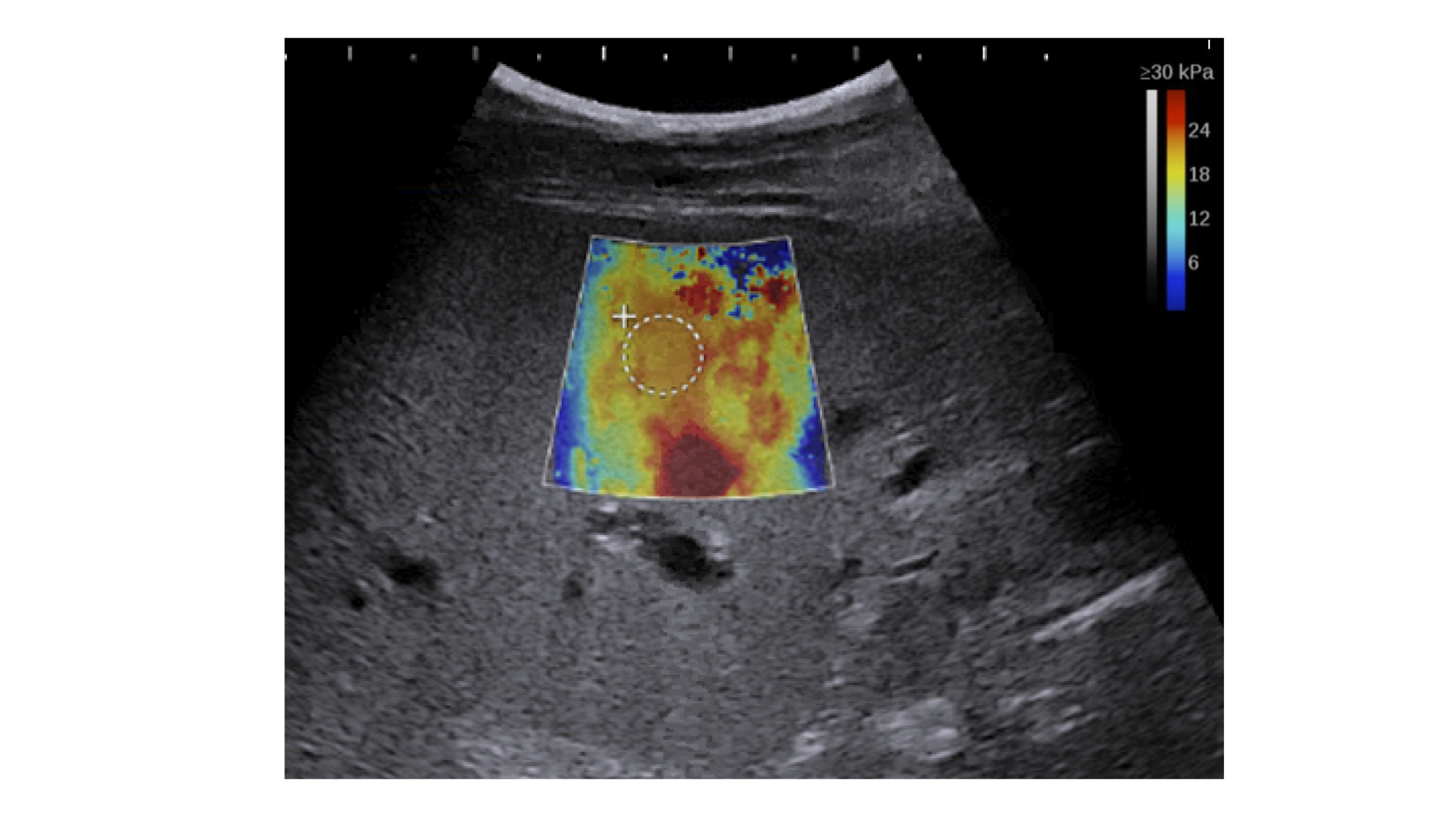

Supplement: Supplementary file 1 — Additional file 1. Supplemental tables and supplemental figure. [file 13244_2021_1090_MOESM1_ESM.docx]
